# Combined inhibition of GLI and FLT3 signaling leads to effective anti-leukemic effects in human acute myeloid leukemia

**DOI:** 10.18632/oncotarget.16304

**Published:** 2017-03-16

**Authors:** Emily-Marie Latuske, Hauke Stamm, Marianne Klokow, Gabi Vohwinkel, Jana Muschhammer, Carsten Bokemeyer, Manfred Jücker, Maxim Kebenko, Walter Fiedler, Jasmin Wellbrock

**Affiliations:** ^1^ Department of Oncology, Hematology and Bone Marrow Transplantation with Section Pneumology, Hubertus Wald University Cancer Center, University Medical Center Hamburg-Eppendorf, Hamburg, Germany; ^2^ Institute of Biochemistry and Signal Transduction, University Medical Center Hamburg-Eppendorf, Hamburg, Germany

**Keywords:** Hedgehog, non-canonical, GLI, FLT3, AML

## Abstract

Activation of the Hedgehog pathway has been implicated in the pathogenesis of several tumor types including myeloid leukemia. Previously we demonstrated that overexpression of Hedgehog downstream mediators GLI1/2 confers an adverse prognosis to patients with acute myeloid leukemia (AML) and is correlated with a FLT3 mutated status. To analyze a possible non-canonical activation of the Hedgehog pathway via FLT3 and PI3K, we performed blocking experiments utilizing inhibitors for FLT3 (sunitinib), PI3K (PF-04691502) and GLI1/2 (GANT61) in FLT3-mutated and FLT3 wildtype AML cell lines and primary blasts. Combination of all three compounds had stronger anti-leukemic effects in FLT3-mutated compared to FLT3 wildtype AML cells *in vitro*. Interestingly, the colony growth of normal CD34^+^ cells from healthy donors was not impeded by the triple inhibitor combination possibly opening a therapeutic window for the clinical use of inhibitor combinations. Besides, combined treatment with sunitinib, PF-04691502 and GANT61 significantly prolonged the survival of mice transplanted with FLT3-mutated MV4-11 cells compared to the single agent treatments. Furthermore, the inhibition of FLT3 and PI3K resulted in reduced GLI protein expression and promotor activity in FLT3-mutated but not in FLT3 wildtype AML cell lines in western blotting and GLI1/2 promoter assays supporting our hypothesis of non-canonical GLI activation via FLT3.

In summary, FLT3-mutated in contrast to FLT3 wildtype cells or normal human hematopoietic progenitor cells are exquisitely sensitive to combined inhibition by FLT3, PI3K and GLI1/2 overcoming some of the limitations of current FLT3 directed therapy in AML. The development of GLI1/2 inhibitors is highly desirable.

## INTRODUCTION

**Figure 1:**
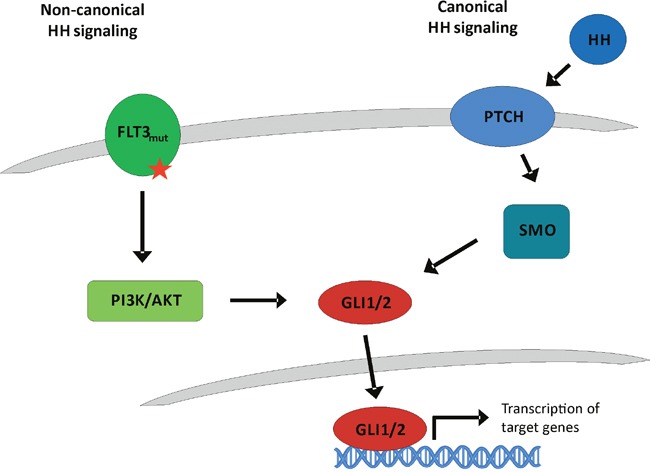
Classical canonical and proposed non-canonical Hedgehog signaling In the classical canonical Hedgehog signaling cascade, Hedgehog ligands bind to the transmembrane receptor Patched-1 (PTCH1). Upon HH ligand binding, PTCH1 is internalized resulting in relief and thus activation of the receptor Smoothened (SMO) which transduces the signal to the GLI transcription factors which regulate cell fate. On the other hand, our proposed non-canonical Hedgehog cascade via FLT3 is HH ligand independent. Due to its ITD or TKD mutation, FLT3 is constitutively activated resulting in an active PI3K and AKT signaling that activates the GLI signaling cascade.

The Hedgehog (HH) signaling pathway is an important stem cell pathway. It plays a role in embryonic as well as adult stem cells [1, 2]. Mammalian cells express 3 different Hedgehog ligands: Sonic (SHH), Desert (DHH) and Indian (IHH) Hedgehog which bind to the transmembrane receptor Patched-1 (PTCH1). Without presence of ligands, PTCH1 represses activation of the transmembrane protein Smoothened (SMO). Upon HH ligand binding, PTCH1 is internalized resulting in relief and thus activation of SMO which transduces the signal to the family of Glioma (GLI) zinc finger transcription factors GLI1, GLI2 and GLI3. GLI1 and GLI2 predominantly represent transcriptional activators while GLI3 acts as transcriptional repressor (Figure 1) [3].

Besides its physiological role, aberrant HH signaling is involved in tumorigenesis. HH pathway activation has been shown to be involved in different solid human cancers including glioblastoma, medulloblastoma or basal cell carcinoma [4], as well as in the progression of hematological malignancies like chronic myelogeneous leukemia (CML) or multiple myeloma [5–7].

Recently, we could show that expression of HH pathway transcription factors GLI1 and GLI2 represents a negative prognostic marker for patients with AML and targeted inhibition of GLI1/2 mediates anti-leukemic effects *in vitro* and *in vivo*. Interestingly, the impact of GLI2 expression on the AML patients’ survival was correlated to the occurrence of mutant FMS-like tyrosine kinase 3 (FLT3) [8]. We therefore proposed an interaction between the FLT3 pathway and the HH mediators GLI1/2.

Recent studies indicate a role of ligand- and SMO-independent non-canonical HH signaling via phosphoinositide 3-kinase (PI3K)/AKT as shown in renal cell carcinoma and esophageal adenocarcinoma or TGFβ/SMAD in pancreatic cancer [9–11].

Activating FLT3 mutations still confer an adverse prognosis to the affected AML patients. Recently, combination therapy including the FLT3 inhibitor midostaurin and standard chemotherapy has shown to result in a survival benefit compared to chemotherapy alone [12]. Nevertheless, about 30 % of patients are still refractory to induction chemotherapy including a FLT3 inhibitor such as midostaurin, sorafinib or sunitinib [13–15, 12]. An additional 30 % of patients relapse after achieving an initial complete remission [12].

Activated FLT3 signals downstream via the PI3K/AKT axis [16]. Interestingly, Riobò et al. identified a crosstalk between HH and PI3K signaling [17]. Thus we wondered whether combined inhibition of the FLT3, PI3K and GLI1/2 axis may increase anti-leukemic effects in AML.

## RESULTS

### Anti-proliferative effects upon combined inhibition of GLI, FLT3 and PI3K

**Figure 2:**
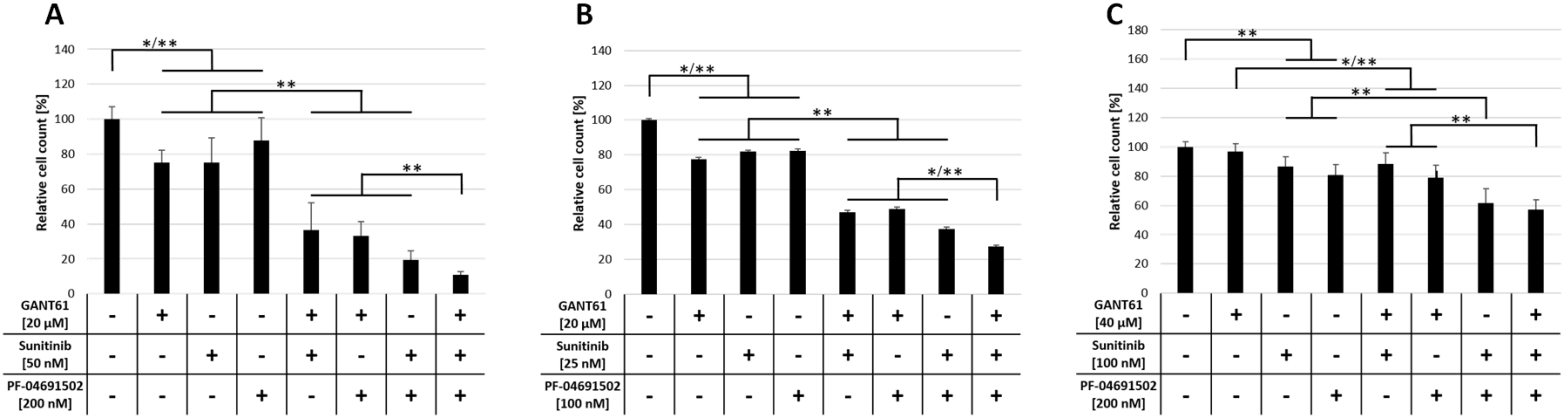
Anti-proliferative effects upon combined inhibition of GLI, FLT3 and PI3K in AML cell lines MV4-11 **(A)**, MOLM-13 **(B)** and OCI-AML5 **(C)** cells were treated with the indicated concentrations of GANT61, sunitinib and PF-04691502 alone or in combination of two or all three agents or DMSO as solvent control and the effect on the proliferation capacity was analyzed after three days by determining viable cell counts using the cell viability analyzer Vi-Cell ™ XR. Error bars represent the mean values ± standard deviation from at least three independent experiments. * *P*<0.05, ** *P*<0.001 in the Welch's *t*-test.

In this study we aimed to analyze a possible non-canonical activation of GLI-mediated HH signaling via the FLT3/PI3K axis in AML. To investigate therapeutic efficacy of a combined inhibition of FLT3, PI3K and GLI on growth of AML cells, the FLT3-mutated AML cell lines MV4-11 and MOLM-13, as well as FLT3 wildtype cell line OCI-AML5 were treated with different concentrations of the FLT3 inhibitor sunitinib, the PI3K inhibitor PF-04691502 and the GLI inhibitor GANT61 either alone or in combination of two or all three agents. The effect on proliferation was analyzed by determining viable cell counts. Of note, in preliminary proliferation experiments, MOLM-13 showed a higher sensitivity towards sunitinib and PF-04691502 treatment compared to MV4-11 while OCI-AML5 cells had the lowest sensitivity to all three compounds (data not shown). Therefore, we adjusted the concentrations accordingly. All single agent treatments of MV4-11 or MOLM-13 cells reduced viable cell counts statistically significant compared to the solvent control (Figure 2A and 2B). Double agent treatments resulted in a further statistically significant reduction of proliferation capacity compared to single agent treatments. Triple agent treatment led to an additional statistically significant higher impairment of proliferation compared to double agent treatments (Figure 2A and 2B). Anti-proliferative effects upon GLI, FLT3 and PI3K inhibition in FLT3 wildtype OCI-AML5 cells occurred only at higher concentrations and to a lower extend compared to FLT3-mutated MV4-11 and MOLM-13 cells (Figure 2C). For single agents, only sunitinib and PF-04691502 treatments reduced cell counts statistically significant compared to the solvent control. In double agent treatments of OCI-AML5 cells, only the combination of sunitinib with PF-04691502, but not with GANT61 had an additional and statistically significant inhibitory effect compared to the respective single agent treatments. Again, the combination of all three compounds led to the most pronounced anti-proliferative effect which was statistically significant compared to the GANT61 + sunitinib and GANT61 + PF-04691502 double agent treatments (Figure 2C).

**Figure 3:**
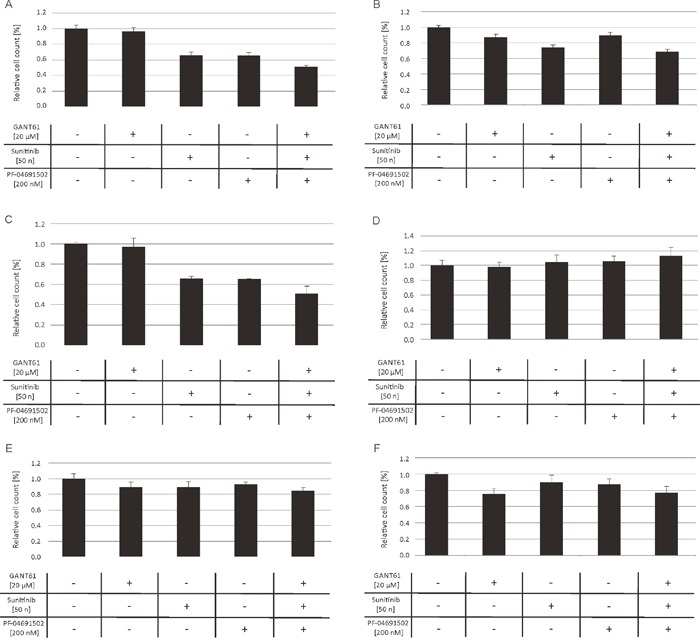
Anti-proliferative effects upon combined inhibition of GLI, FLT3 and PI3K in primary AML blasts AML blasts from FLT3-mutated **(A-C)** and FLT3 wildtype patients **(D-F)** were treated with GANT61, sunitinib and PF-04691502 alone, the triple agent combination or DMSO as solvent control. The effect on the proliferation capacity was analyzed after three days by determining viable cell counts using the cell viability analyzer Vi-Cell™ XR.

Furthermore, we investigated the impact of combined inhibition of GLI, FLT3 and PI3K in primary AML blasts of FLT3-mutated (n=5) as well as FLT3 wildtype AML patients (n=12). The AML blasts were treated with low concentrations of the FLT3 inhibitor sunitinib, the PI3K inhibitor PF-04691502 and the GLI inhibitor GANT61 either alone or with the triple combination of agents. As expected, the FLT3-mutated AML blasts were more responsive to the triple inhibitor treatment. In four of five analyzed cases (80%), the combined inhibition of GLI, FLT3 and PI3K resulted in a decreased or at least slightly decreased cell growth compared to the single agent treatment. On the other hand, only four of twelve FLT3 wildtype cases (33%) showed increased responsiveness upon combined treatment with GANT61, sunitinib and PF-04691502 (see representative data of FLT3-mutated and FLT3 wildtype AML blasts in Figure 3).

### Apoptosis induction upon combined inhibition of GLI, FLT3 and PI3K

The AML cell lines MV4-11, MOLM-13 and OCI-AML5 were investigated for apoptosis induction upon combined GANT61, sunitinib and PF-04691502 treatment. MV4-11 and MOLM-13 cells were treated with 20 μM GANT61, 50 nM sunitinib and 200 nM PF-04691502 while OCI-AML5 cells were treated with higher compound concentrations (40 μM, 100 nM and 200 nM, respectively). Compounds were used in single, double and triple treatments of all agents and apoptosis rates were determined by flow cytometry.

**Figure 4:**
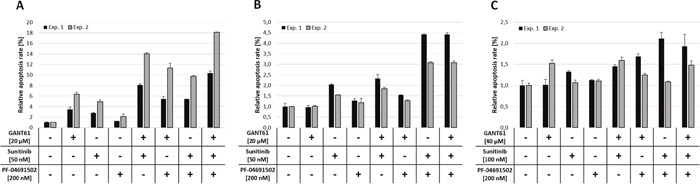
Apoptosis induction upon combined inhibition of GLI, FLT3 and PI3K MV4-11 **(A)**, MOLM-13 **(B)** and OCI-AML5 **(C)** cells were treated with the indicated concentrations of GANT61, sunitinib and PF-04691502 alone or in combination of two or all three agents or DMSO as solvent control and induction of apoptosis was measured after 48h by flow cytometry using Annexin V and propidium iodide. Error bars represent the mean values ± standard deviation from two independent experiments.

In MV4-11 cells, all three compounds induced apoptosis. Combinations of two inhibitors were more effective compared to the single agent treatments and the triple inhibitor combination had the most pronounced effect on apoptosis rates (Figure 4A). Combined inhibition of MOLM-13 cells by GLI, FLT3 and PI3K also resulted in apoptosis induction but less effective compared to MV4-11 cells (Figure 4B). In OCI-AML5 cells, combined inhibition of GLI, FLT3 and PI3K did not induce any noticeable apoptosis, even at high compound concentrations (Figure 4C).

### Reduced colony formation capacity of FLT3-mutated AML cells upon combined inhibition of GLI, FLT3 and PI3K

Leukemic cells, similar to hematopoietic stem and progenitor cells, possess the ability to grow colonies in semisolid media. We wanted to analyze if combined inhibition of GLI, FLT3 and PI3K impairs the colony formation capacity of AML cell lines. To assess any inhibitory effects on normal hematopoietic progenitor cells we included CD34^+^ cells from healthy donors. MV4-11, MOLM-13 and OCI-AML5 cells as well as CD34^+^ cells were treated with combinations of GANT61 (10 μM), sunitinib (25 nM) or PF-04691502 (500 nM) and the effect on the colony formation capacity was examined.

**Figure 5:**
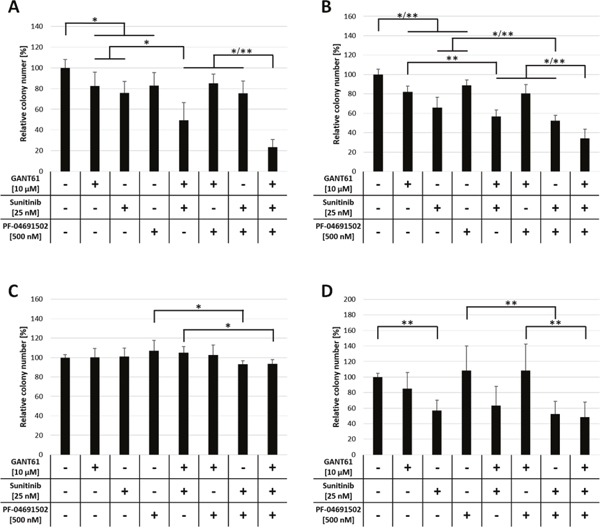
Reduced colony formation capacity of FLT3-mutated AML cells upon combined inhibition of GLI, FLT3 and PI3K Cell lines MV4-11 **(A)**, MOLM-13 **(B)** and OCI-AML5 **(C)** as well as CD34^+^ progenitor cells **(D)** (*n* = 3) were incubated with single, double and triple combinations of GANT61, sunitinib and PF-04691502 or DMSO control in the indicated concentrations and cultured in methylcellulose-based semi-solid media. The number of colonies was counted after 5 to 10 days. Error bars represent the mean values ± standard deviation from at least two independent experiments. * *P*<0.05, ** *P*<0.001 in the Welch's *t*-test.

In MV4-11 and MOLM-13 cells, single agent treatment with all three compounds reduced the colony numbers statistically significant compared to the solvent control. Of double agent treatments, only the combination of GANT61 and sunitinib reduced the colony numbers significantly compared to the single agent treatments. The most dramatic and statistically significant impact on colony formation was observed upon triple agent treatment (Figure 5A, 5B). In FLT3 wildtype OCI-AML5 cells, all treatment combinations had merely minimal effects on colony growth (Figure 5C). In CD34^+^ cells, neither single agent treatment with GANT61 or PF-04691502 nor the combination of both substances had a significant effect on the colony formation (Figure 5D). In contrast, single agent treatment with sunitinib reduced the colony growth to 56.8 ± 13.5 % which was statistically significant compared to the solvent control. Interestingly, no further reduction of colony numbers was observed upon combination of sunitinib with GANT61 or PF-04691502.

### Decreased GLI1 protein expression upon sunitinib treatment

**Figure 6:**
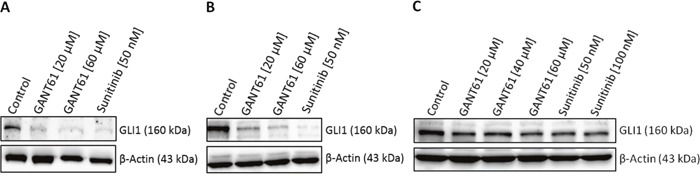
Decreased GLI1 protein expression upon sunitinib treatment Cell lines MV4-11 **(A)**, MOLM-13 **(B)** and OCI-AML5 **(C)** were treated with the indicated concentrations of GANT61, sunitinib or DMSO as solvent control for three days. GLI1 protein expression was examined by western blot analysis. One representative western blot from two independent experiments is shown.

To analyse the effects of FLT3 inhibition on HH signaling downstream targets, the FLT3-mutated AML cell lines MV4-11 and MOLM-13 as well as the FLT3 wildtype AML cell line OCI-AML5 were treated with 50 nM and 100 nM sunitinib, respectively, and western blot analysis for GLI1 as active HH signaling readout was performed (Figure 6). As a control for GLI inhibition, the cells were treated with GANT61 in two to three different concentrations ranging from 20 μM to 60 μM. In both FLT3-mutated cell lines, sunitinib treatment decreased GLI1 protein expression equally compared to the GLI specific inhibitor GANT61 indicating an interaction between the FLT3 and HH pathway (Figure 6A and 6B). As expected, in the FLT3 wildtype cell line OCI-AML5, GANT61 and sunitinib treatment decreased GLI1 protein expression only moderately and to a lesser extend compared to the FLT3-mutated AML cell lines (Figure 6C).

### Decreased GLI promotor activity upon combined inhibition of GLI, FLT3 and PI3K

**Figure 7:**
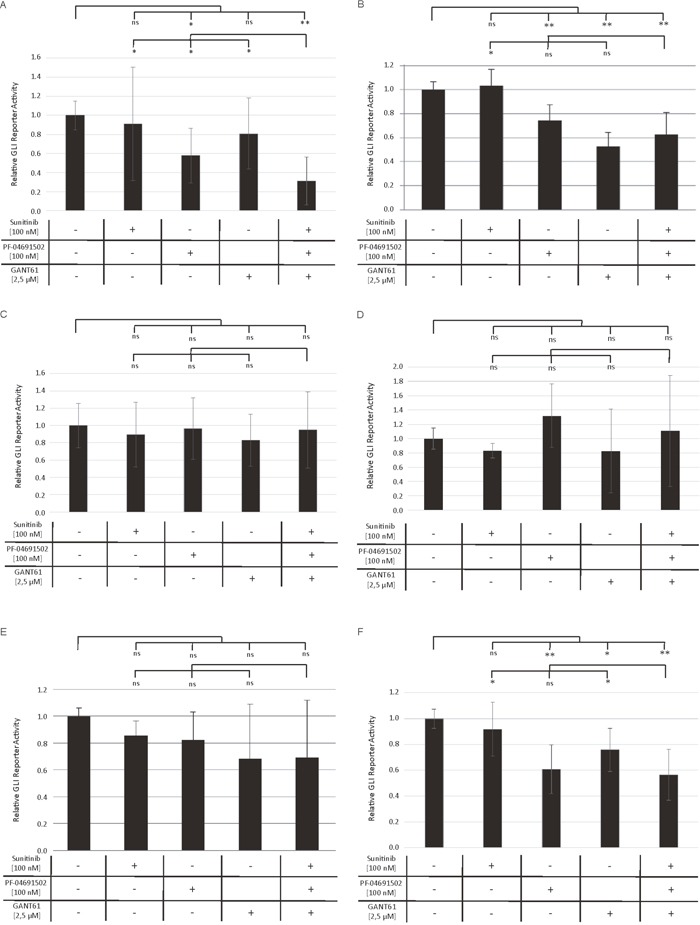
Decreased GLI promoter activity upon combined inhibition of GLI, FLT3 and PI3K Stable GLI Reporter AML cells lines MV4-11 **(A)**, MOLM-13 **(B)**, OCI-AML3 **(C)**, OCI-AML5 **(D)**, IMS-M2 **(E)** and HL60 **(F)** were treated with sunitinib, PF-04691502 and GANT61 or the triple agent combination. The GLI promoter activity was measured after 24h using the Dual-GLO Luciferase Assay Kit and the Infinite F200 PRO reader. The firefly luciferase-mediated GLI promoter activity was normalized to the renilla luciferase-mediated CMV promoter activity. Error bars represent the mean values ± standard deviation from at least three independent experiments. * *P*<0.05, ** *P*<0.001 in the Welch's *t*-test; ns signifies statistically not significant.

In order to prove that the observed increased anti-leukemic effects upon combined inhibition of GLI, FLT3 and PI3K are mediated via inhibition of the GLI cascade, we produced several AML cell lines expressing the luciferase gene under the control of GLI transcriptional response elements which allows a direct monitoring of the GLI promotor activity. The FLT3-mutated AML cell lines MV4-11 and MOLM-13 as well as FLT3 wildtype cell lines OCI-AML3, OCI-AML5, IMS-M2 and HL60 were treated with low concentrations of sunitinib, PF-04691502, GANT61 or the triple combination. As expected, we observed the most prominent effects on GLI promotor activity in the FLT3-mutated AML cell lines MV4-11 and MOLM-13. The inhibitor combination resulted in a highly significant decrease compared to the untreated cells (Figure 7A and 7B). Furthermore in MV4-11 cells, this reduction was also significantly stronger compared to the single agent treatment (Figure 7A), while the effect of GANT61 or PF-04691502 alone was not exceeded by the triple combination in MOLM-13 cells (Figure 7B). In the FLT3 wildtype cell lines OCI-AML3, OCI-AML5 and IMS-M2 neither the single agent treatments nor the triple combination resulted in significantly reduced GLI promoter activity using low inhibitor concentrations (Figure 7C-7E). The FLT3 wildtype cells HL60 were more responsive to the treatment with GANT61 and PF-04691502. No further decrease of the GLI promoter activity could be observed in the triple combination compared to single agent treatment as the PI3K inhibitor PF-04691502 alone had a strong effect which might be due to the NRAS mutation resulting in activated downstream signaling via PI3K in HL60 cells (Figure 7F) [18].

### Non-canonical GLI signaling predominates in AML

**Figure 8:**
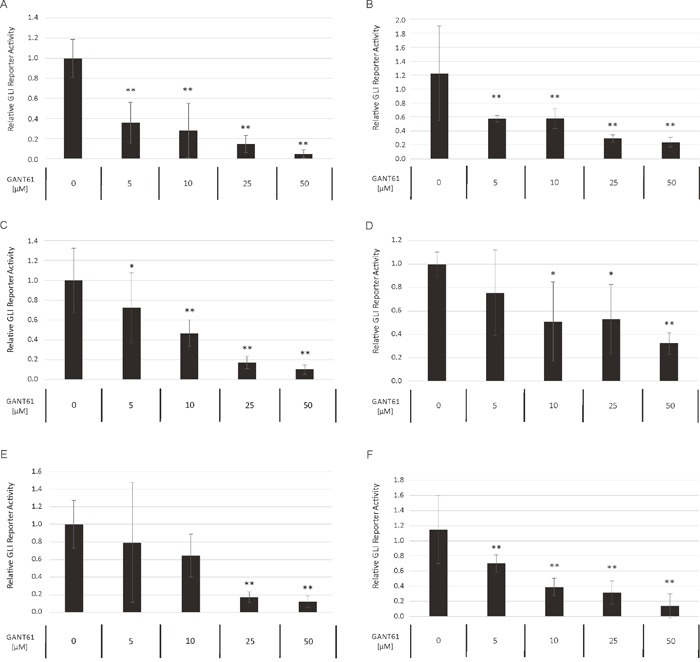
Dose-dependent reduction of GLI promoter activity upon GANT61 treatment The AML Reporter cell lines MV4-11 **(A)**, MOLM-13 **(B)**, OCI-AML3 **(C)**, OCI-AML5 **(D)**, IMS-M2 **(E)** and HL60 **(F)** were treated with increasing GANT61 concentrations or DMSO as solvent control. The GLI promoter activity was measured after 24h using the Dual-GLO Luciferase Assay Kit and the Infinite F200 PRO reader. The firefly luciferase-mediated GLI promoter activity was normalized to the renilla luciferase-mediated CMV promoter activity. Error bars represent the mean values ± standard deviation from at least three independent experiments. *P<0.05, ** *P*<0.001 in the Welch's *t*-test.

**Figure 9:**
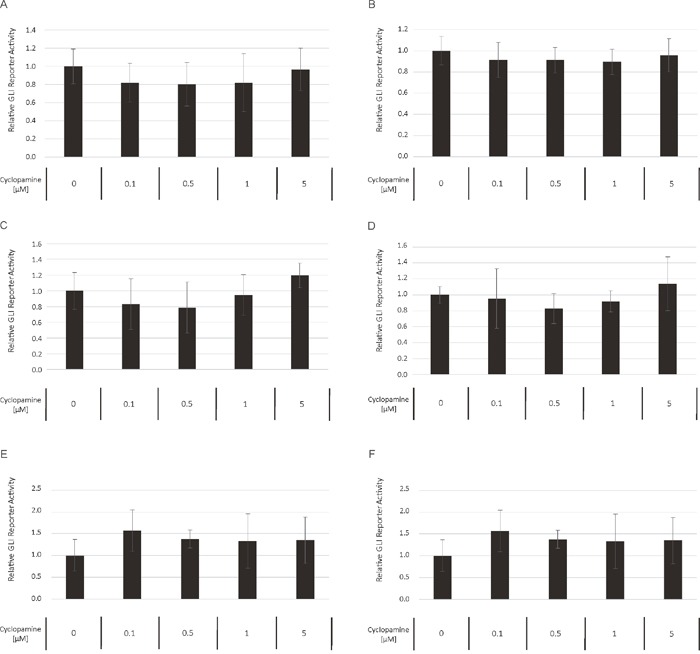
Cyclopamine treatment has no impact on GLI promoter activity The AML Reporter cell lines MV4-11 **(A)**, MOLM-13 **(B)**, OCI-AML3 **(C)**, OCI-AML5 **(D)**, IMS-M2 **(E)** and HL60 **(F)** were treated with increasing cyclopamine concentrations. The GLI promoter activity was measured after 24h using the Dual-GLO Luciferase Assay Kit and the Infinite F200 PRO reader. The firefly luciferase-mediated GLI promoter activity was normalized to the renilla luciferase-mediated CMV promoter activity. Error bars represent the mean values ± standard deviation from at least three independent experiments.

To compare the relative preponderance of canonical vs. non-canonical HH signaling in AML, we analyzed the impact of GANT61 as direct GLI inhibitor to the SMO inhibitor cyclopamine with regard to their ability to block the GLI promoter activity in a reporter assay. Several AML cell lines were treated with increasing concentrations of GANT61 and cyclopamine starting with their published IC50 concentration as provided by the manufacturer, respectively. Although to a different extend, the treatment with GANT61 led to a dose-dependent reduction of the GLI promoter activity in all analyzed AML cell lines (Figure 8A-8F). But surprisingly, no decrease of the GLI promoter activity could be observed upon treatment with cyclopamine in the analyzed cell lines (Figure 9A-9F).

### Prolonged survival in a mouse model upon combined inhibition of GLI, FLT3 and PI3K

**Figure 10:**
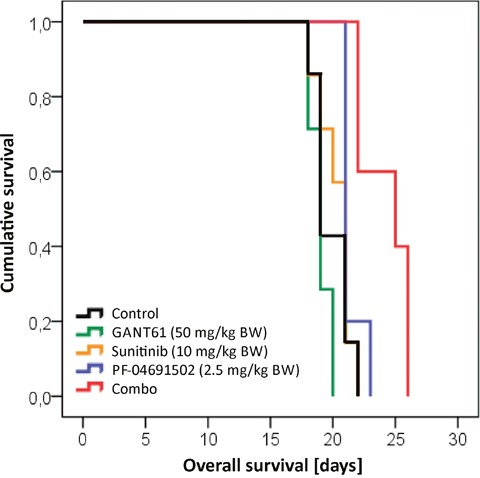
Prolonged survival in a mouse model upon combined inhibition of GLI, FLT3 and PI3K NSG mice were intravenously transplanted with MV4-11 cells and treated with GANT61 (50 mg/kg BW; n=7; green line), sunitinib (10 mg/kg BW, n=7; orange line), PF-04691502 (2.5 mg/kg BW; n=5; blue line), the triple combination (red line; n=7) or solvent control (black line; n=7). Only the triple combination could significantly prolong the survival compared to the control group (p=0.004). Furthermore, the survival advantage of the triple combination was statistically significant better in comparison to all three single agent treatments (p=0.001 for GANT61, p=0.004 for sunitinib and p=0.020 for PF-04691502, respectively). BW = body weight.

In order to investigate the potential of a combined inhibition of GLI, FLT3 and PI3K in FLT3-mutated AML *in vivo*, NSG mice were transplanted with MV4-11 cells and treated with GANT61, sunitinib, PF-04691502, their combination or DMSO as a control. As shown in Figure 10, the combined treatment with all substances could prolong the animals’ survival while no survival advantage could be observed upon single agent treatment.

## DISCUSSION

Based on our previous work, we hypothesized that the HH pathway might be activated in a non-canonical fashion via FLT3 in AML, especially in patients with activating mutations [8]. Therefore the combined inhibition of GLI, FLT3 and PI3K could represent a promising treatment option for AML patients. Accordingly, we analyzed two FLT3-mutated as well as a FLT3 wildtype AML cell line. Upon combined inhibition by sunitinib, PF-04691502 and GANT61, stronger anti-leukemic effects could be observed in the FLT3-mutated AML cell lines MV4-11 and MOLM-13 compared to the FLT3 wildtype AML cell line OCI-AML5. Additionally, the effects on OCI-AML5 cells occurred only at higher compound concentrations. Similar results were obtained with primary blasts from AML patients as the combined treatment of GLI, FLT3 and PI3K had more prominent effects on blasts from FLT3-mutated compared to FLT3 wildtype AML patients. Furthermore in a leukemia mouse model of MV4-11, the combined treatment with GANT61, sunitinib and PF-04691502 could prolong the animals’ survival. This observation demonstrates that the non-canonical FLT3/GLI axis is of pathophysiological importance in FLT3-mutated AML (see Figure 1 for schematic representation). Moreover, we could show that inhibition of FLT3 signaling has a direct impact on GLI protein expression in the FLT3-mutated AML cell lines using western blot analysis and GLI reporter assays. Recent work by Lim and colleagues underlines our findings as they could demonstrate that crosstalk between FLT3 signaling and GLI2 promotes myeloid leukemogenesis [19]. An additional report suggests that crosstalk between GLI1 and PI3K/AKT/NF-κB renders AML cells resistant to radiation [20]. Non-canonical upregulation of the GLI transcription factors has been described for a number of oncogenic pathways including TGF-β, RAS/RAF/MEK/MAPK, ERBB2, PI3K/AKT and mTOR [9–11, 21–23]. We could recently show that GLI1 is activated via ERBB2 and PI3K/AKT/mTOR in esophageal adenocarcinoma in a SMO-independent manner [10]. Another group could demonstrate a non-canonical activation of GLI1 in esophageal adenocarcinoma via TNF-α and subsequently mTOR/S6K1 [21] while Zhou and colleagues could recently show that GLI1 and GLI2 are non-canonically activated via PI3K/AKT in human renal cell carcinoma [9]. Using GLI Reporter cell lines, we could show that an active GLI signaling cascade in AML cells is mainly SMO-independent as the treatment with the SMO inhibitor cyclopamine had hardly any impact on the GLI promoter activity of all six analyzed AML cell lines. On the other hand, the direct GLI inhibitor GANT61 resulted in a dose-dependent reduction of the GLI promoter activity underlining our hypothesis of a non-canonical GLI signaling axis in AML cells. It was recently shown that in comparison to healthy human hematopoietic cells, AML cells have a reduced expression of primary cilia which are required for functional canonical Hedgehog signaling [24]. These data further emphasize the importance of the non-canonical Hedgehog pathway activation in AML.

The non-canonical activation could be mediated via different pathways as was shown here for the FLT3 cascade in the FLT3-mutated AML cells. In other AML cell lines, the activation of the GLI cascade is probably mediated via other non-canonical pathways. In the AML cell line HL60, treatment with the PI3K inhibitor PF-04691502 had also a marked impact on the GLI reporter activity. These effects might be due to the fact that HL60 cells carry a NRAS mutation leading to activated PI3K downstream signaling which renders the cells sensitive to PI3K inhibition [18]. Non-canonical GLI signaling via RAS and PI3K has already been described in other cancer entities [23, 25] and might also be responsible for active GLI signaling in HL60 cells.

Several potent FLT3 inhibitors have already been tested in early-stage clinical trials for their efficacy as single agents in FLT3-mutated AML [26–29]. Unfortunately, despite achievement of blast free states or partial responses, the clinical effects remain transient, possibly due to resistance mechanisms such as protection within the bone marrow niche and emergence of resistant clones [30]. A multi-targeted inhibition of both, FLT3 and HH signaling, could lower the risk of development of therapy resistance by simultaneously targeting the leukemic blasts at different levels and thereby lowering the chance for the survival of resistant clones. Such an approach could decrease the risk of relapse after the treatment. Furthermore, combined therapy could allow a reduction of inhibitor administration. This could decrease adverse side effects due to high dosage that is often required to achieve clinical efficacy [31]. In the present study, the *in vitro* clonogenic growth of CD34^+^ cells from healthy donors was inhibited by sunitinib treatment. Interestingly, neither the addition of GANT61 or PF-04691502 nor their combination had an additional effect on the CD34^+^ cells’ colony number. This finding could open a therapeutic window for a combination of FLT3 inhibitors with GLI inhibitors in the treatment of FLT3-mutated AML.

To date, some FLT3 inhibitors have already been tested in clinical trials in combination with conventional chemotherapy [32–35]. In a recently published randomized double blind clinical trial with the multi-targeted tyrosine kinase inhibitor midostaurin, an improvement of overall survival of FLT3-mutated AML patients compared to placebo was demonstrated [12]. Sorafenib, another multi-targeting tyrosine kinase inhibitor, has been tested in combination with chemotherapy in two randomized, placebo-controlled phase II trials in AML patients, showing variable benefit for the patients [13, 14]. Sunitinib has recently been tested in a phase I/II clinical trial in combination with intensive chemotherapy in FLT3-mutated AML patients [15]. In spite of high remission rates, 20-30 % of the patients did not respond to the treatment. This subset of FLT3-mutated patients that does not respond to initial chemotherapy combined with FLT3 inhibitors could potentially benefit from combined targeting of FLT3 and downstream signaling proteins. For instance, the mTOR inhibitor RAD001 enhanced anti-leukemic effects of sunitinib in AML cell lines and primary samples [36] and concomitant blockade by sunitinib and the MEK/ERK inhibitor AZD6244 induced synergistic inhibitory effects in FLT3-mutated AML cell lines and primary samples [37]. Based on our previously published and present data, HH signaling, possibly non-canonically activated via FLT3, represents another promising target for combined therapy with FLT3 inhibitors.

Several HH inhibitors have been tested in clinical studies for the treatment of different cancer entities, with the focus being mainly on SMO inhibitors [38]. SMO inhibitors such as vismodegib, IPI-926 and sonidegib showed high efficacy in cancer types like basal cell carcinoma or medulloblastoma that often harbor activating mutations in the HH pathway. So far, vismodegib and sonidegib have been approved by the US Food and Drug Administration for the treatment of advanced basal cell carcinoma. In 2011, first promising results of a small phase I study with the SMO inhibitor PF-04449913 in hematologic malignancies including AML were published [39] and two studies with PF-04449913 in patients with AML and myelodysplastic syndrome (MDS) or acute leukemia, respectively, are currently recruiting participants (NCT01546038, NCT01841333). A phase II study with sonidegib in acute leukemia has been completed but the results are still awaited (NCT01826214) while, disappointingly, a phase II study with vismodegib in patients with AML and MDS had to be prematurely terminated due to lower-than-expected efficacy (NCT01880437).

Development of resistance against vismodegib has already occurred in patients with basal cell carcinoma and medulloblastoma [40–42]. Mechanisms of resistance could be associated with SMO mutations and amplification of GLI2, underlining the importance of this transcription factor for sustained tumor growth [43–45]. Interestingly, upregulation of the PI3K pathway was found as a resistance mechanism against a SMO antagonist in medulloblastoma, indicating a clinically relevant interaction of both pathways [46]. Therefore, a combined inhibition of HH signaling and the non-canonically activating pathways emerges as a promising cancer therapy approach given the high incidence of non-canonical HH pathway activation in mechanisms underlying the development and progression of human cancers.

As the potential underlying reason for therapy resistance and relapse in AML patients, the leukemic stem cell (LSC) has emerged as a target therefore representing an important topic of investigation. Similar to the hierarchy in normal hematopoiesis, leukemia is believed to originate from LSCs which are thought to be resistant to chemotherapy and responsible for disease relapse [47, 48]. Besides indications for a correlation between aberrantly activated HH-GLI activity in cancer stem cells in various cancer entities [49–52], HH-GLI appears to be also implicated in LSC biology. Recent studies could demonstrate a role of deregulated HH signaling and GLI transcription factors in the maintenance, function and drug resistance of myeloid LCSs whose leukemia promoting capacities could get effectively diminished by HH pathway inhibition [53–55]. Interestingly, HH pathway inhibition sensitized the myeloid progenitor cells for chemotherapy and tyrosine kinase inhibitors [53, 55], underlining the importance of combined therapy approaches with HH inhibitors in the treatment of AML.

As both the canonical and non-canonical HH signaling cascade converge at the level of the HH transcription factors, the inhibition of GLI appears to be the most reasonable target in the HH pathway. Importantly, overexpression of GLI1 and/or GLI2 could be correlated with a poor prognosis for the patients in several cancer entities (e.g. [8, 56–60]). So far, only a few GLI inhibitors have been developed, including, HPI1-4, GANT58 or GANT61 with the latter being the most potent agent [61]. The selective GLI1/2 inhibitor GANT61 has shown high anti-cancer efficacy in *in vitro* studies as well as in animal models but only few data exist on the compounds’ pharmacokinetics [62]. Therefore, continuing efforts are needed to develop GLI inhibitors that can be safely translated into clinical use and could be combined to target FLT3/PI3K/GLI in the treatment of FLT3-mutated AML patients.

## MATERIALS AND METHODS

### Cell lines and cell culture

The cell lines used in this study were either purchased recently at the DSMZ (Deutsche Sammlung von Mikroorganismen und Zellkulturen GmbH) or authenticated by the Multiplex human Cell Authentication test (Multiplexion). The human AML cell lines MV4-11, MOLM-13, HL60 and IMS-M2 were cultured in RPMI-1640 medium (Gibco) supplemented with 10 % fetal bovine serum (FBS, Biochrom GmbH). The human AML cell line OCI-AML3 was cultured in α-MEM medium (Gibco) supplemented with 20 % FBS. The human AML cell line OCI-AML5 was cultured in α-MEM medium (Gibco) supplemented with 20 % FBS and 10 ng/mL GM-CSF (PeproTech GmbH). Primary AML blasts and CD34^+^ progenitor cells from healthy donors were cultured in IMDM medium (Gibco) supplemented with 10 % FBS, 10 % horse serum (Gibco), and 10^−6^ M hydrocortisone (Sigma-Aldrich). All cells were maintained in a humidified incubator with 5 % CO_2_ at 37°C.

### Enrichment of CD34^+^ progenitor cells from healthy donors

CD34^+^ progenitor cells were isolated from the mononuclear cell fraction of leukapheresis products of G-CSF primed healthy, anonymous donors using the indirect CD34 MicroBead Kit and the VarioMACS Separator (Miltenyi Biotec). The purity of CD34^+^ cells ranged between 93 % and 97 % as determined by flow cytometry.

### Proliferation assay

FLT3-mutated AML cell lines MV4-11 and MOLM-13, the FLT3 wildtype OCI-AML5 and primary AML blasts were cultured with different concentrations of the GLI inhibitor GANT61 (2,20-[[Dihydro-2-(4-pyridinyl)-1,3(2H,4H)-pyrimidinediyl]bis(methylene)]bis[N, N-dimethylbenzenamine]; Tocris Bioscience), the FLT3 inhibitor sunitinib (LC Laboratories) and the PI3K inhibitor PF-04691502 (2-Amino-8-[trans-4-(2-hydroxy-ethoxy)cyclohexyl]-6-(6-methoxy-3-pyridinyl)-4-methyl-pyrido[2,3-d]pyrimidin-7(8H)-one; Tocris Bioscience) either alone or in combination of two or all three agents or dimethyl sulfoxide (DMSO, Sigma-Aldrich) as solvent control. Cell numbers were determined after three days using the cell viability analyzer Vi-Cell ™ XR (Beckman Coulter) in at least three independent experiments.

### Apoptosis assay

AML cell lines were treated with different concentrations of GANT61, sunitinib and PF-04691502 either alone or in combination of two or all three agents or DMSO control. In two independent experiments, induction of apoptosis was measured after 48 hours by flow cytometry using Annexin V (MabTag GmbH) and propidium iodide (Invitrogen). FACS analysis was performed using the FACSCalibur with CellQuestPro Software (BD Biosciences).

### Colony formation assay

AML cell lines and CD34^+^ progenitor cells were seeded with single, double and triple combinations of GANT61, sunitinib and PF-04691502 or DMSO control in Methocult (Methocult H4230 for AML cell lines and Methocult H4435 Enriched for CD34^+^ cells, Stemcell Technologies). The number of colonies was counted after 5 to 10 days using an inverted microscope (Axiovert 25, Zeiss).

### GLI Reporter assays

Stable GLI Reporter AML cell lines were produced using lentiviral particles containing the firefly luciferase gene under the control of GLI transcriptional response elements and as internal control the renilla luciferase gene under CMV promoter elements (Cignal™ Lenti Reporters, Qiagen) followed by puromycin and hygromycin selection. Stable GLI Reporter cells were treated with different small molecule inhibitors and the GLI promoter activity was measured after 24h using the Dual-GLO Luciferase Assay Kit (Promega) and the Infinite F200 PRO reader (Tecan). The firefly luciferase-mediated GLI promoter activity was normalized to the renilla luciferase-mediated CMV promoter activity.

### Protein isolation and western blot analysis

Proteins of MV4-11, MOLM-13 and OCI-AML5 cells were extracted using the trichloroacetic acid method. Protein concentration was determined using the DC Protein Assay (Bio-Rad). For each sample a total of 20 μg protein was separated using a 4-20 % or 4-12 % tris-glycine SDS-polyacrylamide gel (Thermo Fisher Scientific). The protein was transferred to a nitrocellulose membrane and the membrane was incubated with either rabbit anti-human GLI1 (C68H3, 1:1000, Cell Signaling Technology) or mouse anti-human β-ACTIN (sc-47778, 1:5000, Santa Cruz Biotechnology) at 4°C over night. HRP-linked anti-rabbit IgG (7074S, 1:10.000) and anti-mouse IgG (NXA931, 1:10.000) secondary antibodies were purchased from Cell Signaling Technology and GE Healthcare, respectively. Membranes were incubated with secondary antibodies for 1h at room temperature. Imaging was performed using the Amersham ECL Prime Western Blotting Detection Reagent (GE Healthcare) and the Fusion SL imaging system with Fusion software (Vilber Lourmat).

### Xenograft model

5 × 10^5^ MV4-11 cells were injected intravenously into NSG mice (NOD.Cg-Prkdcscid Il2rgtm1Wjl/SzJ). Mice received a daily oral dose of 50 mg/kg GANT61, 10 mg/kg sunitinib, 2,5 mg/kg PF-04691502, the triple agent combination or solvent control. Mice were sacrificed when showing clear symptoms of leukemia. Leukemic infiltration of peripheral blood, bone marrow and spleen was confirmed by flow cytometry.

### Statistical analysis

Data from the *in vitro* assays and *in vivo* mouse model were statistically analyzed by the Welch's *t-*test or Kaplan Meier survival analysis including the log rank test using SPSS 17 (SPSS Inc.), respectively. A *P* value less than 0.05 was considered to be statistically significant.
